# Изменение функции яичников и овариального резерва после комбинированного лечения дифференцированного рака щитовидной железы

**DOI:** 10.14341/probl13635

**Published:** 2026-05-20

**Authors:** М. О. Корчагина, Е. Н. Андреева, М. С. Шеремета, Г. А. Мельниченко

**Affiliations:** Национальный медицинский исследовательский центр эндокринологии им. академика И.И. Дедова; Endocrinology Research Centre; Национальный медицинский исследовательский центр эндокринологии им. академика И.И. Дедова; Институт клинической медицины; Endocrinology Research Centre; Moscow State University of Medicine and Dentistry of A.I. Evdokimov

**Keywords:** дифференцированный рак щитовидной железы, терапия радиоактивным йодом, осложнения, функция яичников, антимюллеров гормон, овариальный резерв, differentiated thyroid cancer, radioactive iodine therapy, complications, ovarian function, ovarian reserve, anti-Müllerian hormone

## Abstract

**ОБОСНОВАНИЕ:**

ОБОСНОВАНИЕ. Парадигма комбинированного лечения дифференцированного рака щитовидной железы (ДРЩЖ) существует с середины прошлого столетия и включает тиреоидэктомию и терапию радиоактивным йодом (РЙТ), после которых назначается супрессивная терапия. Комбинированное лечение улучшает прогноз, особенно у пациентов с высоким риском рецидива ДРЩЖ, однако может быть сопряжено с развитием различных осложнений, в том числе со стороны женской репродуктивной системы.

**ЦЕЛЬ:**

ЦЕЛЬ. Оценка и сравнительный анализ функции яичников и овариального резерва (ОР) с использованием антимюллерова гормона (АМГ), фолликулостимулирующего гормона (ФСГ), лютеинизирующего гормона (ЛГ), эстрадиола (Е2) и эстрона (Е1) у женщин репродуктивного возраста, получивших комбинированное лечение по поводу ДРЩЖ, и у здоровых женщин той же возрастной группы.

**МАТЕРИАЛЫ И МЕТОДЫ:**

МАТЕРИАЛЫ И МЕТОДЫ. В одноцентровом одномоментном сравнительном исследовании проанализированы клинико-морфологические, анамнестические и лабораторные параметры у пациенток, прошедших тиреоидэктомию и один курс РЙТ по поводу ДРЩЖ, и у здоровых женщин.

**РЕЗУЛЬТАТЫ:**

РЕЗУЛЬТАТЫ. В исследование включено 97 женщин в возрасте от 18 до 40 лет: 67 женщин с ДРЩЖ с медианой возраста 31 год [26; 36], прошедших комбинированное лечение по поводу заболевания, а также 30 здоровых женщин в группу сравнения с медианой возраста 30 лет [28; 35].

Частота нарушения менструального цикла составила 33% у пациенток с ДРЩЖ и 13% у здоровых женщин. При сравнении результатов гормонального обследования не выявлено различий в уровнях ФСГ, ЛГ, ПРЛ, Е1 и Е2 между группами. Уровень АМГ стал единственным параметром, значимо отличающимся у пациенток с ДРЩЖ и у здоровых женщин — 2,49 нг/мл [1,1; 3,3] и 3,6 нг/мл [2,62; 4,18] соответственно (Р<0,004). У 18 (27%) пациенток с ДРЩЖ уровень АМГ был ниже 1,2 нг/мл, в группе здоровых женщин — в одном случае. Предикторами снижения уровня АМГ<1,2 нг/мл стали возраст женщины на момент РЙТ и возраст на момент обследования на фоне супрессивной терапии после комбинированного лечения, с помощью индекса Юдена определены пороговые значения в 31 год и 33 года соответственно.

**ЗАКЛЮЧЕНИЕ:**

ЗАКЛЮЧЕНИЕ. Уровень АМГ значимо ниже у пациенток с ДРЩЖ, прошедших комбинированное лечение, по сравнению со здоровыми женщинами той же возрастной группы, при этом возраст на момент РЙТ 31 год и старше, а также возраст на момент обследования 33 года и старше ассоциированы с низким ОР после комбинированного лечения ДРЩЖ.

## Abstract (en)

**BACKGROUND:**

BACKGROUND: The paradigm of combined treatment for differentiated thyroid cancer has been in use since the mid-20th century. This approach involves thyroidectomy, followed by radioactive iodine therapy, and after patients may be prescribed suppressive therapy. Combined treatment improves outcomes, particularly in cases of high risk of recurrence. However, it can also lead to a range of secondary complications, including those affecting the reproductive system.

**AIM:**

AIM: Assessment and comparative analysis of ovarian function and ovarian reserve (OR) using anti-Müllerian hormone (AMH), follicle-stimulating hormone (FSH), luteinizing hormone (LH), prolactin (PRL), estradiol (E2) and estrone (E1) in the early follicular phase in women of reproductive age who received combined treatment for DTC, and in healthy women of the same age.

**MATERIALS AND METHODS:**

MATERIALS AND METHODS. In a single-center, comparative cross-sectional study, clinical and morphological, anamnestic and laboratory parameters were analyzed in patients who underwent thyroidectomy and one course of radioiodine therapy for DTC, and in healthy women.

**RESULTS:**

RESULTS: The study enrolled 90 women aged 18 to 40 years: 67 women with DTC with a median age of 31 years [26; 36] who underwent combined treatment, and 30 healthy women with a median age of 30 years [28; 35]. The frequency of menstrual cycle disorders over the past year was 33% in women with DPT and 13% in healthy women.

When comparing the results of hormonal examination, it was revealed that the levels of FSH, LH, PRL, E1 and E2 did not differ significantly between the groups. The level of AMH was the only parameter that significantly differed in patients with DTC receiving combined treatment and in healthy women — 2.49 ng/ml [1.1; 3.3] and 3.6 ng/ml [2.62; 4.18], respectively (P < 0.004). In 18 (27%) patients with DTC, the level of AMH was < 1.2 ng/ml, in the group of healthy women low level of AMH was found only in one case. Only patient's age at the time of RAIT and age at the time of examination on the background of thyroid stimulating hormone suppression were associated with diminished ovarian reserve, using the Juden index, the cut-off points of 31 years and 33 years were determined, respectively.

**CONCLUSION:**

CONCLUSION. The level of AMH in patients with DTC who undergo combined treatment is significantly lower compared to healthy women of the same age. Only patient's age at the time of RAIT and age at the time of examination on the background of thyroid stimulating hormone suppression were associated with diminished ovarian reserve.

## ОБОСНОВАНИЕ

Дифференцированный рак щитовидной железы (ДРЩЖ) — наиболее распространенное злокачественное новообразование щитовидной железы (ЩЖ), составляющее 90–95% от всех вариантов рака ЩЖ, с соотношением мужчин и женщин ~1:4 [1]. Часть пациентов проходит комбинированное лечение ДРЩЖ, включающее тиреоидэктомию (ТЭ) и терапию радиоактивным йодом (РЙТ), после которых при отсутствии противопоказаний назначается супрессивная терапия. Послеоперационная РЙТ рекомендуется в соответствии с установленным риском рецидива заболевания — при высоком риске она показана всем пациентам, при промежуточном назначается в индивидуальном порядке, а в случае низкого риска, как правило, не проводится [2–4]. При таком подходе важным становится не только оценка пользы терапии в отношении общей и безрецидивной выживаемости пациентов, но и прогнозирование потенциальных вторичных осложнений с целью проведения их профилактики, а также своевременного привлечения специалистов смежных областей в случае их развития.

В связи с распространенностью ДРЩЖ у женщин детородного возраста, влияние проводимой терапии на функциональное состояние яичников, репродуктивное здоровье и течение беременности пристально изучается в ретроспективный и проспективных исследованиях. На сегодняшний день обсуждается роль как неоднократной смены тиреоидного статуса (эутиреоз-гипотиреоз-субклинический тиреотоксикоз) в процессе комбинированного лечения и последующего наблюдения, так и непосредственного влияния РЙТ на функцию яичников и овариальный резерв (ОР) пациенток с ДРЩЖ [5–7]. Зафиксировано развитие транзиторной олигоменореи/аменореи, а также снижение ОР после РЙТ по данным лабораторной и инструментальной диагностики, однако долгосрочное влияние на фертильность не доказано [8][9].

## ЦЕЛЬ ИССЛЕДОВАНИЯ

Оценить функцию яичников и ОР у женщин репродуктивного возраста, получивших комбинированное лечение по поводу ДРЩЖ, и у здоровых женщин, соотносимых по возрасту.

## МАТЕРИАЛЫ И МЕТОДЫ

## Место и время проведения исследования

Место проведения. Исследование проведено в ГНЦ РФ ФГБУ «НМИЦ эндокринологии им. академика И.И. Дедова» Минздрава России (далее — НМИЦ эндокринологии) отделении радионуклидной терапии.

Время исследования. Исследование проводили с 2023 г. по 2024 гг.

## Изучаемые популяции (одна или несколько)

Целевые популяции определялись критериями включения и невключения.

Пациентки с ДРЩЖ

Критерии включения: женщины; возраст от 18 до 40 лет включительно; ДРЩЖ (МКБ-10 — С73), установка диагноза произведена в ходе планового патологоанатомического исследования операционного материала; проведение комбинированного лечения ДРЩЖ, включающего ТЭ и один курс РЙТ; длительность супрессивной терапии после РЙТ — 12–18 мес.

Критерии невключения: только хирургическое лечение ДРЩЖ; более 1 курса РЙТ; супрессивная терапия >18 мес или <12 мес; установленный диагноз бесплодия; операции на яичниках или лучевая терапия на органах малого таза в анамнезе; синдром поликистозных яичников; беременность; лактация; прием комбинированных оральных контрацептивов (КОК) на момент обследования или завершение приема КОК позднее чем за 2 мес до начала обследования; прием заместительной гормональной терапии (ЗГТ) половыми стероидами или завершение приема ЗГТ позднее чем за 2 мес до начала обследования.

Способ формирования выборки — сплошной.

Пациентки с ДРЩЖ в ходе анализа были разделены на подгруппы в зависимости от уровня АМГ — подгруппа пациенток с нормальным уровнем АМГ (≥1,2 нг/мл) и со сниженным уровнем АМГ (<1,2 нг/мл), что соответствует низкому ОР.

Здоровые женщины

Критерии включения: женщины; возраст от 18 до 40 лет включительно.

Критерии невключения: диагностированное доброкачественные или злокачественные заболевания ЩЖ; установленный диагноз бесплодия; операции на яичниках или лучевая терапия на органах малого таза в анамнезе; синдром поликистозных яичников; беременность; лактация; прием КОК на момент обследования или завершение приема КОК позднее чем за 2 мес до начала обследования; ЗГТ половыми стероидами или завершение приема ЗГТ позднее чем за 2 мес до начала обследования.

Способ формирования выборки — произвольный.

Дизайн исследования — одномоментное сравнительное исследование.

## Методы

После оперативного лечения диагноз ДРЩЖ устанавливался по результатам патологоанатомического исследования в НМИЦ эндокринологии. Проводилась послеоперационная стратификация риска рецидива заболевания с выделением трех групп (группа низкого риска, группа промежуточного риска, группа высокого риска) на основании рекомендаций Американской тиреоидологической ассоциации (American Thyroid Association, 2015 г.) для определения дальнейшей тактики ведения — наблюдение или направление на РЙТ [10][11].

Всем пациенткам с ДРЩЖ и здоровым женщинам проведены общеклинический осмотр, изучение анамнеза, текущего статуса и жалоб с установкой соответствия критериям. В рамках исследования проведен гормональный анализ, включающий определение антимюллерова гормона (АМГ), фолликулостимулирующего гормона (ФСГ), лютеинизирующего гормона (ЛГ), пролактина (ПРЛ) в сыворотке крови с использованием автоматизированной тест-системы VITROS 3600 (Ortho Clinical Diagnostics), а также эстриола (Е1), эстрадиола (E2) с помощью высокоэффективной жидкостной хроматографии/масс-спектрометрии (ВЭЖХ/МС). Забор крови проводился утром с 09:00 до 11:00 натощак на 3–5‑й дни менструального цикла однократно. Лабораторные исследования проводили на базе клинико-диагностической лаборатории и лаборатории метаболомных и протеомных исследований НМИЦ эндокринологии.

Нарушение менструального цикла устанавливалось в соответствии с российскими клиническими рекомендациями «Аномальные маточные кровотечения» 2021 г., «Аменорея и олигоменорея» 2021 г. [12][13]. Сниженный ОР устанавливался при уровне АМГ менее 1,2 нг/мл (Poseidon criteria, клинические рекомендации «Женское бесплодие» 2021 г.) [14][15].

## Статистический анализ

Статистический анализ выполнен с помощью программы Statistica 13.0 (Statsoft, USA), для проведения ROC-анализа использована программа IBM SPSS Statistics v.26 (IBM Corporation, США). Описательная статистика количественных признаков представлена с помощью медиан, первых и третьих квартилей (Me [Q1; Q3]), категориальных признаков — с помощью абсолютных и относительных частот (n (%)). Сравнительный анализ двух независимых групп по количественным признакам выполнен с помощью критерия Манна-Уитни (U-тест), по категориальным признакам — с помощью двустороннего точного критерия Фишера (ТКФ2). Для оценки прогностической ценности параметров и поиска пороговых значений был выполнен ROC-анализ, пороговые значения выбирались согласно индексу Юдена. Для пороговых значений были рассчитаны операционные характеристики: диагностическая чувствительность (ДЧ), диагностическая специфичность (ДС), прогностическая ценность положительного результата (ПЦПР) и прогностическая ценность отрицательного результата (ПЦОР) с 95% доверительными интервалами (ДИ) с помощью онлайн-калькулятора (https://statpages.info/ctab2x2.html).

Уровень статистической значимости принят равным 0,05. При множественных сравнениях уровень статистической значимости скорректирован с помощью поправки Бонферрони (Р0), при этом значения Р, превышающие порог статистической значимости, но <0,05, интерпретировались как статистическая тенденция.

## Этическая экспертиза

Работа одобрена локальным этическим комитетом при ГНЦ РФ ФГБУ «НМИЦ эндокринологии» Минздрава России. Протокол заседания локального этического комитета №18 от 12.10.2022 г.

## РЕЗУЛЬТАТЫ

Размер выборки основной группы составил 67 пациенток с ДРЩЖ (папиллярная карцинома ЩЖ в 100% случаев), получивших комбинированное лечение по поводу заболевания, средний возраст пациенток на момент исследования — 31 год [ 26; 36]. В группу сравнения было включено 30 здоровых женщин, средний возраст составил 30 лет [ 28; 35]. Сравнительная характеристика групп по клинико-анамнестическим параметрам представлена в таблице 1, по результатам лабораторного исследования — в таблице 2.

**Table:** Таблица 1. Сравнительный анализ клинико-анамнестических данных у пациенток с ДРЩЖ (N=67) и здоровых женщин (N=30) Поправка Бонферрони Р0=0,05/13=0,004¹ U-test² ТКФ2

Признак	Пациентки с ДРЩЖ	Контроль	P
N	Me [ Q1; Q3] /n (%)	N	Me [ Q1; Q3] /n (%)
Возраст, годы	67	31 [ 26; 36]	30	30 [ 28; 35]	0,854¹
Индекс массы тела, кг/м²	67	23,4 [ 20,1; 27,6]	30	22,4 [ 20,5; 23,2]	0,314¹
Менархе, годы	67	13 [ 12; 13]	30	12 [ 11; 13]	0,339¹
НМЦ за последний год	67	22 (33%)	30	3 (13%)	0,051²

**Table:** Таблица 2. Сравнительный анализ результатов гормонального обследования у пациенток с ДРЩЖ (N=67) и здоровых женщин (N=30) Поправка Бонферрони Р0=0,05/6=0,008

Признак	Пациентки с ДРЩЖ	Здоровые женщины	P
N	Me [ Q1; Q3] /n (%)	N	Me [ Q1; Q3] /n (%)
ФСГ, Ед/л	67	5,1 [ 4,5; 6,9]	30	5 [ 4,6; 5,7]	0,207
ЛГ, Ед/л	67	4,7 [ 3,2; 6,8]	30	4,75 [ 4,1; 5,7]	0,612
Пролактин, мЕд/л	67	320,8 [ 251,7; 507,9]	30	394,55 [ 303; 492,6]	0,312
АМГ, нг/мл	67	2,49 [ 1,1; 3,3]	30	3,6 [ 2,62; 4,18]	0,003
Е1, пмоль/л	67	109 [ 92; 136]	30	104 [ 90; 130]	0,435
E2, пмоль/л	67	149 [ 102; 198]	30	158 [ 125; 191]	0,301

Не было обнаружено статистически значимых различий по возрасту, частоте нарушения менструального цикла (НМЦ), индексу массы тела (ИМТ), фракциям эстрогенов, а также ФСГ, ЛГ, ПРЛ между группами. Единственным значимо отличающимся параметром стал уровень АМГ, который ниже у пациенток с ДРЩЖ — 2,49 нг/мл [ 1,1; 3,3] по сравнению с 3,6 нг/мл [ 2,62; 4,18] у здоровых женщин, Р<0,003. Графическое отображение уровней АМГ представлено на рисунке 1.

**Figure:**
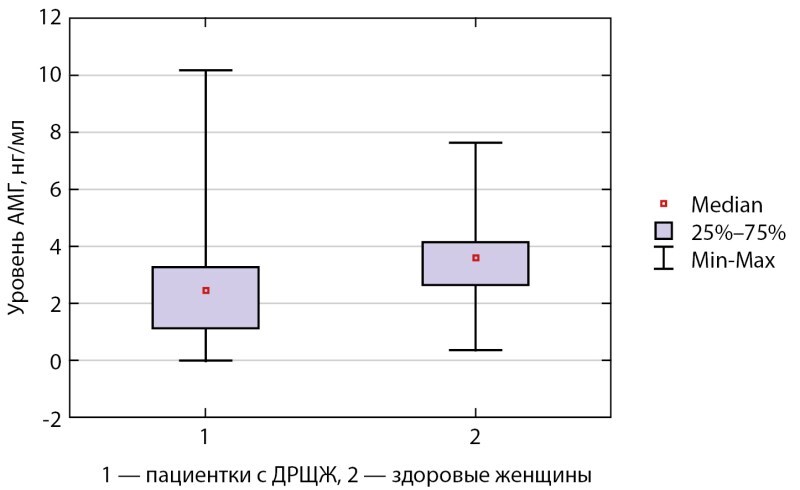
Рисунок 1. Уровень АМГ у пациенток с ДРЩЖ и здоровых женщин.

С целью выявления причин более низких значений АМГ нами был проведен сравнительный анализ внутри основной группы в зависимости от нормального и сниженного АМГ, общая характеристика пациенток с ДРЩЖ и результаты сравнительного анализа представлены в таблицах 3 и 4 соответственно.

**Table:** Таблица 3. Общая характеристика пациенток с ДРЩЖ

Признак	N	Me [ Q1; Q3] /n (%)
Возраст на момент обследования, годы	67	31 [ 26; 36]
Возраст на момент РЙТ, годы	67	30 [ 25; 35]
Менархе, годы	67	13 [ 12; 13]
ИМТ, кг/м²	67	23,1 [ 20,1; 27]
ИМТ	Дефицит	67	8 (12%)
Норма	31 (46%)
Избыточная масса тела	18 (27%)
Ожирение	10 (15%)
Кумулятивная активность, МБк	67	3760 [ 3500; 3850]
Длительность супрессивной терапии после РЙТ, мес	67	12 [ 12; 16]
Стадия 1	67	67 (100%)
Подготовка к РЙТ	Тироген	67	21 (31%)
Отмена	46 (69%)
ФСГ, Ед/л	67	5,1 [ 4,5; 6,9]
ЛГ, Ед/л	67	4,7 [ 3,2; 6,8]
АМГ, нг/мл	67	2,49 [ 1,1; 3,3]
E1, пмоль/л	67	109 [ 92; 136]
E2, пмоль/л	67	149 [ 102; 198]
ПРЛ, Ед/л	67	320,8 [ 251,7; 507,9]
Уровень АМГ<1,2 нг/мл (сниженный ОР)	67	18 (27%)
НМЦ в течение последних 18 мес	67	22 (33%)

**Table:** Таблица 4. Сравнительный анализ результатов гормонального обследования у пациенток с ДРЩЖ с уровнем АМГ≥1,2 нг/мл (N=49) и <1,2 нг/мл (N=18) Поправка Бонферрони Р0=0,05/13=0,004¹ U-тест² ТКФ2

Признак	АМГ≥1,2 нг/мл	АМГ<1,2 нг/мл	p
N	Me [ Q1; Q3] /n (%)	N	Me [ Q1; Q3] /n (%)
Менархе, годы	49	13 [ 12; 13]	18	12 [ 12; 13]	0,330¹
Возраст на момент обследования, годы	49	28 [ 25; 32]	18	35 [ 34; 39]	<0,001¹
Возраст на момент РЙТ, годы	49	27 [ 24; 31]	18	34 [ 33; 37]	<0,001¹
ИМТ, кг/м²	49	23,1 [ 20,4; 27]	18	24,25 [ 19,5; 27,7]	0,745¹
Кумулятивная активность, МБк	49	3760 [ 3160; 3830]	18	3755 [ 3640; 3910]	0,357¹
Длительность супрессивной терапии, мес	49	12 [ 12; 16]	18	14 [ 12; 18]	0,237¹
ФСГ, Ед/л	49	5 [ 4,4; 6,2]	18	6,75 [ 5,1; 10,4]	0,01¹
ЛГ, Ед/л	49	4,6 [ 3,2; 6,3]	18	5,35 [ 3,4; 7,7]	0,548¹
Пролактин, мЕд/л	49	325,3 [ 268,5; 502,9]	18	310 [ 190,3; 582]	0,656¹
E1, пмоль/л	49	109 [ 92; 131]	18	119 [ 85; 136]	0,983¹
E2, пмоль/л	49	166 [ 116; 202]	18	115 [ 91; 146]	0,013¹
НМЦ	49	11 (22%)	18	11 (61%)	0,007²
Подготовка к РЙТ	Тироген	49	14 (29%)	18	7 (39%)	0,553²
Отмена	35 (71%)	11 (61%)

Выявлено, что только возраст на момент обследования и на момент РЙТ значимо различаются среди пациенток с низким и нормальным уровнями АМГ. Средний возраст на момент обследования и на момент лечения РЙТ в группе низкого АМГ составил 35 лет и 34 года соответственно, а в группе нормального — 28 лет и 27 лет соответственно (Р<0,001 в обоих случаях). На уровне статистической тенденции обнаружены различия по уровням ФСГ, Е2, а также частоте НМЦ (Р=0,01; Р=0,013; Р=0,007 соответственно).

Был проведен ROC-анализ с целью оценки прогностических способностей возраста на момент обследования. ROC-анализ представлен на рисунке 2.

**Figure:**
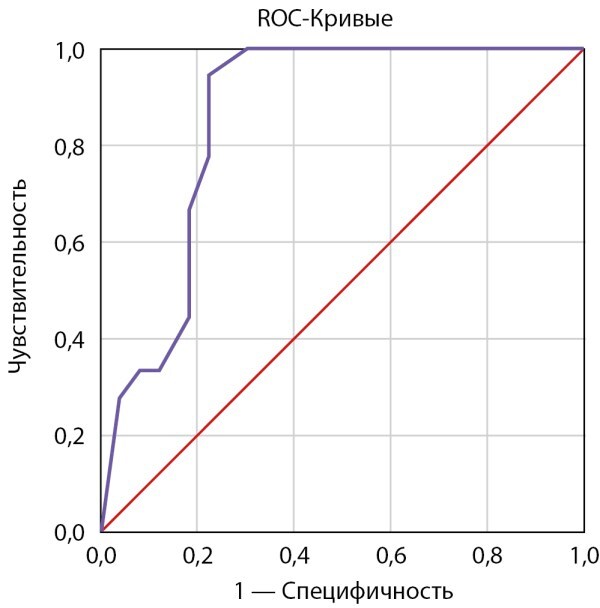
Рисунок 2. ROC-анализ возраста пациенток на момент обследования для прогнозирования уровня АМГ<1,2 нг/мл после комбинированного лечения (N=67).

AUC=0,858 (95% ДИ: 0,771–0,945), что свидетельствует о средней прогностической способности возраста. Пороговое значение, согласно индексу Юдена, равно 33 года. Операционные характеристики порогового значения: ДЧ=0,61 (95% ДИ: 0,44; 0,61), ДС=0,97 (95% ДИ: 0,89; 1,00), ПЦПР=0,94 (95% ДИ: 0,74; 1,00), ПЦОР=0,776 (95% ДИ: 0,70; 0,80).

Таким образом, у пациенток 33 лет и старше вероятность снижения АМГ менее 1,2 нг/мл составляет 77–95%.

Был проведен ROC-анализ с целью оценки прогностических способностей возраста на момент РЙТ. ROC-анализ представлен на рисунке 3.

**Figure:**
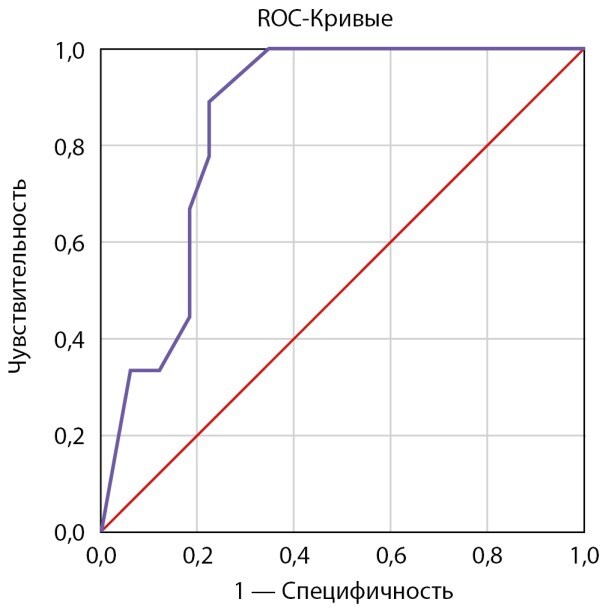
Рисунок 3. ROC-анализ возраста пациенток на момент РЙТ для прогнозирования уровня АМГ<1,2 нг/мл после лечения (N=67).

AUC=0,853 (95% ДИ: 0,764–0,941), что свидетельствует о средней прогностической способности возраста. Пороговое значение, согласно индексу Юдена, — 31 год. Операционные характеристики: ДЧ=0,55 (95% ДИ: 0,43; 0,58), ДС=0,97 (95% ДИ: 0,87; 1,00), ПЦПР=0,94 (95% ДИ: 0,74; 1,00), ПЦОР=0,71 (95% ДИ: 0,64; 0,73).

Таким образом, у пациенток 31 года и старше на момент РЙТ вероятность снижения АМГ менее 1,2 нг/мл составляет 76–93%.

На момент обследования большинство пациенток с ДРЩЖ (91%) не планировали беременность в ближайшие 1–2 года. Только одной пациентке было диагностировано бесплодие, связанное с ановуляцией. Во всей группе средняя активность РЙТ составила 3760 МБк [ 3500; 3850], средняя длительность супрессивной терапии — 12 мес [ 12; 16].

## ОБСУЖДЕНИЕ

Комбинированное лечение ДРЩЖ обладает высокой эффективностью, но может быть сопряжено с развитием различных вторичных осложнений как после ТЭ и РЙТ, так и на фоне длительной супрессивной терапии. Установлено, что смена тиреоидного статуса может быть одним из факторов, влияющим на фолликулогенез и стероидогенез, при этом как гипотиреоз, так и субклинический тиреотоксикоз могут сопровождаться НМЦ с развитием аномальных маточных кровотечений, олигоменореи/аменореи и повышением частоты ановуляторных циклов, а также осложненным течением беременности и негативным влиянием на плод [16][17]. Сама ТЭ, при условии компенсации послеоперационного гипотиреоза, напрямую не сопряжена с изменением функции яичников и снижением ОР, а также не повышает риск осложнений беременности [16][18][19]. Однако Huang N и соавт. установили более низкую частоту зачатия при применении вспомогательных репродуктивных технологий (ВРТ), а также снижение живорождения после тотальной тиреоидэктомии в сравнении с гемитиреоидэктомией [20].

Несмотря на селективность, РЙТ может быть сопряжена с различными вторичными осложнениями в результате реализации радиобиологических эффектов ¹³¹I экстратиреоидно и вне очагов ДРЩЖ. Яичники не накапливают ¹³¹I, но могут получать дозу облучения из крови и близлежащих органов — мочевого пузыря и кишечника, а также от метастазов ДРЩЖ, расположенных в малом тазу и захватывающих ¹³¹I [21]. По результатам работ Ceccarelli и соавт., Rosario и соавт., опубликованных в 2001 и 2006 гг. соответственно, впервые установлена связь между РЙТ и более ранним наступлением менопаузы (P<0,001) [22][23]. Другое наблюдение: НМЦ по типу олигоменореи/аменореи с транзиторным повышением ФСГ (P<0,00001), как правило, в течение первых 12 мес после РЙТ [24–26]. Максимальная частота НМЦ у пациенток с РЩЖ составила 31,1% случаев по результатам работы Sioka и соавт., а в контрольной (здоровые женщины) — только в 14,5% (P<0,02) [24].

В нашей работе мы сравнили клинико-анамнестические параметры и результаты лабораторной диагностики у 67 пациенток с ДРЩЖ и 30 здоровых женщин. Оценка наступления менопаузы у пациенток с ДРЩЖ не проводилась в связи с краткосрочным (до 18 мес) периодом наблюдения после РЙТ и одномоментным видом исследования, однако в 33% (22 пациентки из 67) случаев зафиксировано НМЦ, в подавляющем большинстве — по типу олигоменореи (18 пациенток из 22).

В качестве чувствительного лабораторного маркера для оценки ОР используется АМГ, секретируемый растущими — преантральными и малыми антральными — фолликулами. Определение уровня АМГ полезно для оценки гонадотоксичности различных методов лечения и проведения мер по сохранению фертильности, в особенности это актуально для пациенток репродуктивного возраста с онкологическими заболеваниями [27][28]. АМГ является ранним маркером снижения ОР, которое наблюдается раньше нарушения функции яичников по мере их возрастных изменений с повышением фолликулостимулирующего гормона (ФСГ) и снижением эстрадиола (Е2) [29]. Несмотря на стабильный уровень АМГ во время цикла, его оценка представляет сложности ввиду отсутствия международного стандарта диагностики, однако с распространением автоматизированных анализаторов межлабораторная вариабельность может быть снижена [30]. Уровень АМГ варьируется в зависимости от возраста, при этом как таковых возрастных норм не существует, а вот стремительное снижение АМГ может наблюдаться и при регулярном менструальном цикле или транзиторных его нарушениях [31]. В качестве другого маркера ОР применяют подсчет количества антральных фолликулов (КАФ) в ходе ультразвукового исследования органов малого таза (УЗИ ОМТ), однако нельзя не сказать, что этот метод оценки ОР зависит от технических возможностей и профессионализма специалистов, что влияет на его диагностическую ценность [32].

Первая работа, направленная на изучение ОР с использованием АМГ в когорте пациенток с ДРЩЖ, была проведена только в 2016 г. Оказалось, что уровень АМГ у пациенток с ДРЩЖ, получивших РЙТ, ниже, чем у здоровых женщин, — 2,50 нг/мл и 2,92 нг/мл соответственно (Р<0,038) [25]. Дальнейшие проспективные исследования установили значимое снижение АМГ после РЙТ с наименьшим значением через 3 мес после лечения и лишь частичное его восстановление к концу периода наблюдения [5][26][33][34]. Предикторы снижения — возраст старше 35 лет (Р=0,01), раннее менархе (Р=0,03) и сопутствующий тиреоидит Хашимото (Р=0,015) [26][33].

В нашем исследовании также обнаружен значимо более низкий уровень АМГ у пациенток с ДРЩЖ при сравнении со здоровыми женщинами того же возраста (Р=0,003). Сниженный ОР (АМГ<1,2 нг/мл) выявлен в 27% случаев (18 пациенток) в основной группе по сравнению с 3,3% (одна женщина 35 лет) в контрольной. Основным предиктором снижения АМГ<1,2 нг/мл у пациенток с ДРЩЖ стали более старший возраст на момент проведения РЙТ и на момент обследования, с помощью ROC-анализа и критерия Юдена установлены пороговые значения в 31 год и 33 года соответственно. При этом уровни ФСГ, ЛГ, ПРЛ, Е1 и Е2 значимо не отличались между группами, что показывает ранний характер изменений, не свойственный для преждевременной недостаточности яичников или перименопаузы, но отражающий снижение преантральных и малых антральных фолликулов предположительно из-за интенсификации процессов атрезии и/или изменения процессов фолликулогенеза под влиянием комбинированного лечения ДРЩЖ у части пациенток. Обращает на себя внимание, что на уровне статистической тенденции существует разница в уровнях ФСГ и Е2 (Р=0,01 и Р=0,013 соответственно), а также частоте НМЦ (Р=0,007), что соотносится с морфофункциональными изменениями, наблюдаемыми в яичниках при сниженном ОР [35]. К сожалению, судить о качестве ооцитов с помощью лабораторных или инструментальных маркеров не представляется возможным [36].

Степень влияния РЙТ по поводу ДРЩЖ на организм может зависеть от подготовки к данному этапу лечения и от получаемой пациентом активности ¹³¹I. Отмена тиреоидных гормонов сроком на 4 недели приводит к развитию гипотиреоза со снижением клиренса ¹³¹I, что повышает риск различных осложнений, включая одного из наиболее частых — транзиторного сиалоаденита [37]. При использовании человеческого рекомбинантного ТТГ (рчТТГ) отмечена более высокая частота вторичной облитерации слезоотводящих путей [38].

В рассмотренных нами работах не проводилась оценка изменения репродуктивной функции в зависимости от подготовки к РЙТ или об этом не сообщалось, однако можно предположить, что более длительный период выведения ¹³¹I может повышать риск репродуктивных изменений [5][22–26][33][34]. В нашем исследовании 21 пациентка (31%) получала рчТТГ в качестве подготовки к РЙТ, а 46 (69%) находились на отмене левотироксина натрия в течение 4 недель до радионуклидного лечения. Разницы в клинико-лабораторных параметрах в зависимости от метода подготовки к РЙТ выявлено не было. Частота осложнений РЙТ возрастает при активности в 3700 МБк и более, но в случае снижения ОР и НМЦ изменения могут наблюдаться при 1100 МБк [21][33]. В нашей работе не отмечена связь между изменением ОР и активностью ¹³¹I.

Важно отметить, что повышение риска изменений в ЩЖ плода, врожденных пороков развития (ВПР) и невынашивания в случае проведения РЙТ во время невыявленной беременности подтверждают абсолютное противопоказание данного метода лечения во время гестации и как минимум в течение первых 6 мес после терапии [39]. При планировании беременности через 6–12 мес после РЙТ не выявлено увеличения частоты осложненного течения беременности или ВПР плода [40][41]. Страх перед зачатием в течение нескольких лет после РЙТ необоснован, а сама беременность на фоне заболевания, как правило, не приводит к его клинически значимому прогрессированию, но, безусловно, это не отменяет рекомендуемого наблюдения [42][43].

## Клиническая значимость результатов

Клиническая значимость исследования репродуктивной функции у пациенток с ДРЩЖ основана на изменениях, которые возникают в процессе лечения и связаны как тиреоидным статусом, так и с эффектами ¹³¹I вне остаточной ткани ЩЖ и очагов метастазирования ДРЩЖ. Анализ клинико-анамнестических данных, репродуктивных планов и лабораторно-инструментальных маркеров для оценки функции яичников и ОР позволяет определить необходимость консультирования по сохранению фертильности, включая применение методов ВРТ.

## Ограничения исследования

Основными ограничениями исследования являются одномоментный характер, при котором определение АМГ производилось только после комбинированного лечения, и небольшая выборка пациенток с ДРЩЖ. Следующим этапом с целью подтверждения полученных результатов требуется проведение проспективных исследований с определением АМГ до и после РЙТ. Учитывая предсказуемое влияние возраста на уровень АМГ, возможно рассмотреть включение женщин с ДРЩЖ до 35 лет, однако, по нашему мнению, пациентки 35 лет и старше требуют еще более пристального внимания с учетом снижения рождаемости в этой группе и смещения возраста первой беременности на более поздний репродуктивный возраст. Кроме того, небольшой диапазон активности РЙТ не позволяет судить о том, влияет ли доза на развитие осложнений. С нашей точки зрения, полезным может стать введение третьей группы сравнения — пациенток с тиреотоксикозом, которые получают РЙТ в качестве радикального метода лечения. Еще один фактор, который должен быть учтен в будущих работах — степень подавления ТТГ у пациенток с ДРЩЖ. Кроме того, при наличии технических возможностей, проведение фолликулометрии и подсчет КАФ в раннюю фолликулярную фазу позволит более полноценно оценить ОР и исключить дискордантность показателей.

## ЗАКЛЮЧЕНИЕ

У пациенток, получивших комбинированное лечение ДРЩЖ, отмечен более низкий уровень АМГ, чем у здоровых женщин той же возрастной группы. Сниженный ОР потенциально может отразиться на фертильности, успехе методов ВРТ и продолжительности репродуктивного периода. Необходимо накопление клинического опыта в области влияния комбинированного лечения на репродуктивное здоровье. В настоящее время с учетом увеличения возраста первой беременности и повышения частоты бесплодных пар, в том числе за счет идиопатического бесплодия, важно прогнозировать риски низкого ОР, а также учитывать репродуктивные планы пациенток с ДРЩЖ до и в процессе комбинированного лечения.

## ДОПОЛНИТЕЛЬНАЯ ИНФОРМАЦИЯ

Источник финансирования. Данная работа выполнена в соответствии с планом государственного задания. Регистрационный номер 123021000041-6.

Конфликт интересов. Авторы декларируют отсутствие явных и потенциальных конфликтов интересов, связанных с публикацией настоящей статьи.

Участие авторов. Все авторы внесли значимый вклад в проведение исследования и подготовку статьи, прочли и одобрили финальную версию статьи перед публикацией.
